# The Midwife

**Published:** 1915-04

**Authors:** 


### The Midwife.

It is with considerable hesitation and only after consultation that we present a somewhat pessimistic view of this problem. It is obvious that while editorial opinions depend largely upon personal, there may be opinions perfectly proper to the individual which should not be expressed as matters of editorial policy and that, without insincerity, an editor may express in the latter way, opinions which differ more or less from his personal ones.

Pregnancy is, of course, a natural, physiologic process. Practically, however, it presents many points of analogy to disease and may even be said to conform to the type of a disease more perfectly and more frequently that almost any disease in the strict sense. It is caused by a perfectly specific micro-organism, resembling the exclamation point much more closely than the so-called comma bacillus resembles the comma; the etiology so far as the implantation of this germ is concerned, not only resembles that of the three venereal diseases but is much less often liable to exceptional modes of conveyance. In course, duration, critical termination, symptomatology and histologic reaction to the germ, pregnancy is far more regular in conforming to type than any ordinary disease. With very rare exceptions, it leaves marks which warrant retrospective diagnosis more accurately than almost any disease.

These facts may be considered of little practical importance, but it is of importance to note that pregnancy is beset with dangers of complication, of aberrant results, of serious consequences due solely to anomalies of development and latent results of past disease, that it has a direct mortality at the crisis of about 1, possibly 1½ per cent., that it is exceedingly liable to produce secondary results of serious moment, some resulting in chronic invalidism unless surgically relieved, some involving metabolic disturbances of more than passing importance and one deserving particular importance—the increased liability to local development of cancer. It differs from practically every true disease in the danger inherent to anomalies of development and results of disease fully recovered from; in the seriousness of aberrant localizations of the process set up by the micro-organism; in the seriousness of deviations from typic duration, in either direction; in the frequency of serious sequelae after a vestigium that does not produce either death or alarming symptoms; in the predisposition to cancer.

Excepting, on the one hand, a group of rather uncommon acute infections, tetanus, cerebro-spinal meningitis, gas-phlegmon, etc., and, on the other, a group of chronic processes commonly regarded as nearly inevitably fatal or fatal in a majority of well developed cases—cancer, tuberculosis, certain forms of nephritis, cardiac lesions, diabetes, etc.,—we find that pregnancy, labor, the puerperism and the lactation period taken as a unit, ranks in degree of danger, considerably above the average of a large group of mild infections, inflammatory processes and functional disturbances. In regard to direct mortality, it may be regarded as 1-10 to 1-3 as serious as the group of common, serious infections, such as scarlatina, typhoid, variola, etc., but, counting sequelae and including here, the operative mortality rendered inevitable, it is by many held to be of about half this degree of danger.

If we were speaking of any other affection, these premises would lead to only one logical plea, that of individual and public prophylaxis. It is scarcely necessary to state that both ethical and sociologic reasons absolutely preclude such a plea but we feel justified in stating the opinion that PREGNANCY IS OF SUFFICIENT SERIOUSNESS, IN ITS ANALOGY TO DISEASE, TO DEMAND EXPERT MEDICAL SKILL; 1. To determine, in advance, anatomic and pathologic conditions rendering it especially dangerous; 2. Throughout its entire course, after any appreciable reaction has been produced; 3. During labor and the puerperium; 4. During the period of lactation and especially when the question of lactation is being considered; 5. After what, for a disease, would be considered full convalescence, in order to secure diagnosis of and prompt attention to any possible sequelae.

So far as midwives are concerned, the profession is in exactly the same dilemma as in any other problem in which it is confronted with popular custom. Shall we deal with this problem as many feel we should with the social evil: refuse a compromise short of ideal methods, on the ground that such a policy is beneath our dignity and involves us indirectly in the responsibility? Shall we attempt legal control, under the auspices of our professional authorities, and educate midwives as thoroughly as possible, or shall we be deterred by the danger, so well recognized both in this and other problems, of partial training? Shall such methods, if adopted, be directed mainly toward securing midwives as expert as possible, and in particular, shall we demand that midwives shall be virtually trained nurses, or shall we limit our efforts to pointing out danger lines at which medical aid must be summoned—knowing well that such lines cannot always be recognized and that various factors will interfere with the inforcement of any such requirement? Shall we stand firmly on the principle that pregnancy, labor, the puerperium and the care of the young infant demand the most expert attention attempt either legal prohibition of inexpert assistance or shall we merely attempt popular education to secure medical midwifery instead of amateur, professional or more or less specially trained? Either of these courses would meet with much popular opposition and the contention that we were actuated by interested motives.

These questions must be answered with due regard to actual possibilities, not necessarily those of the immediate present but of the near or remote future. Our own opinion may easily be inferred but we feel that the ultimate answer should be based not on theory but on actual experience, in accordance with practical results. Which method will secure the lowest direct and indirect mortality of mother and child, the least incidence of suffering and disease? That method, whatever it may be, should be adopted and no method should be adopted till we are certain that it conforms to these requirements.

